# The fabrication of robust and highly efficient oil–water separation filters *via* the high temperature sintering of silica micropower†

**DOI:** 10.1039/d4ra02534b

**Published:** 2024-04-25

**Authors:** Emma Sadler, Colin R. Crick

**Affiliations:** a Queen Mary University of London, School of Engineering and Materials Science London UK Emma.Sadler@qmul.ac.uk C.Crick@qmul.ac.uk

## Abstract

Superhydrophobic materials have been shown to have many attractive properties, however, their functionality can easily be lost due to the failure of the air layer. For long lasting air layer retention, dedicated mechanisms to maintain this layer and/or reintroduce air into the system are essential. Any air reintroduction control would allow for increased air lifetime but would require a porous material that allows air flow to be effective. Here, we prepared highly porous superhydrophobic materials, fabricated through facile sintering of silica nanoparticles followed by chemical functionalisation. Sintering temperatures were varied to maximise the material's strength and water contact angles, with angles of up to 153° achieved. Furthermore, the porous properties were demonstrated through oil/water separation experiments, where separation efficiencies of up to 98% were recorded.

## Abstract 2

Superhydrophobic materials have been shown to have many attractive properties, however, their functionality can easily be lost due to the failure of the air layer.

## Graphical abstract

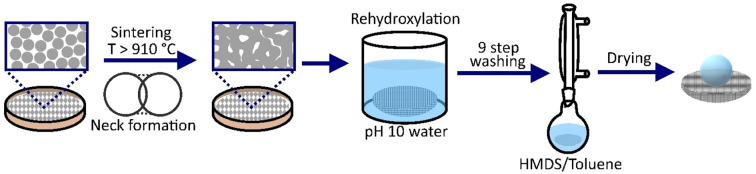

## 1. Introduction

Superhydrophobic surfaces, which show high water contact angles and low contact angle hysteresis, have a range of functionalities due to their water repellent nature. Those that show Cassie–Baxter wetting behaviour have the potential for great impact in submerged environments due to their ability to retain air within the micro-/nano-structures.^1–3^ Though degradation of surface features is often reported as the primary limitation of superhydrophobic materials, in an underwater environment the loss of the thin air layer (also known as the plastron) resulting from external factors (hydrostatic pressure, gas diffusion *etc.*) is of equal concern. Once this air loss occurs (a so-called Cassie–Baxter to Wenzel wetting transition) the surface loses the bulk of its water repelling properties.^4–10^ In applications that are sensitive to surface area, such as water drag reduction or microbial repulsion, this can lead to a worse functional performance than a chemically equivalent smooth surface. The nature of the wetting transition varies between surfaces due to differences in morphology and surface chemistry, where some materials show a near instantaneous transition while others are capable of maintaining air for longer and reaching metastable (partial wetting) states. Many studies have presented hierarchical or re-entrant structures to increase overall lifetime by prolonging the transition.^2,11–15^ Though materials design can slow the transition to a wetted state, the implementation of mechanisms to maintain or reintroduce air to the system ensures the longest plastron lifetime and thus the longest lifetime of properties. One way of achieving this is by using porous superhydrophobic materials and pneumatic air control. Many techniques to prepare such materials described within the literature can be expensive, multi-step or require specialized equipment and reagents. Within, we demonstrate the possibility of using commercially available silica, to produce porous samples *via* a simple sintering pathway. We further demonstrate the materials' functionality through oil/water separation tests in addition to degradation experiments to both physical and chemical conditions.

## 2. Experimental

### 2.1 Materials

Acetone, ammonia, hexamethyldisilzane (HMDS), HCl, 30% hydrogen peroxide solution, KOH, methanol, NaCl, and toluene were all purchased from Sigma-Aldrich Chemical Co. Silica nanoparticles prepared using LUDOX-HS (40% wt SiO_2_) were generously gifted by the Thermo Fisher research and development team.

### 2.2 Silica filter fabrication

**Fig. 1:**
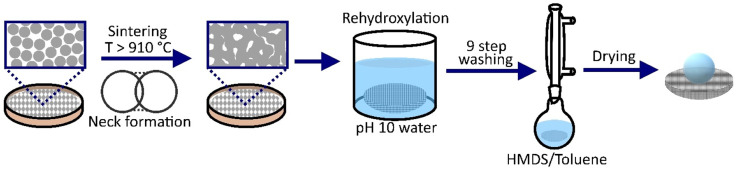
Schematic showing the preparation and functionalisation of porous superhydrophobic materials.

Silica nanoparticles were added to a ceramic crucible and gently tapped to evenly distribute particles. The crucible was placed flat in an oven and heated to a sintering temperature (910–980 °C) for 16 hours at a ramp rate of 1 °C min^−1^. Once cooled the porous samples were rehydroxylated by suspension in DI water (pH10, adjusted with ammonia) for 16 hours. Materials were then washed with DI water (x2), water at pH2 (x1), adjusted with HCl, DI water (x2), methanol (x2) and acetone (x2) before drying at 80 °C in an oven.^16,17^ All samples were then dried in a vacuum oven at 130 °C for 16 hours to ensure the samples were fully dry. Hydrophobically functionalisation of the silica was carried out using hexamethyldisilazane (HMDS, 1 mL), which was added to toluene (400 mL) and stirred for 30 min. Dried samples were added to the solution and refluxed at 120 °C for 24 h. After completion samples were dried in an oven at 80 °C (Fig. 1).

### 2.3 Characterisation

Porous samples were analysed using an FEI Inspect F SEM operating at an acceleration voltage of 5–10 kV. Before visualisation, samples were vacuum sputter coated in a thin layer (∼5 nm) of gold to improve electrical conductivity inside the SEM. Fourier Transform Infrared (FTIR) measurements were taken using a Bruker Tensor 27 FTIR spectrometer over a range of 400 to 4000 cm^−1^. Static water contact angle measurements were taken using an Ossila Contact Angle Goniometer using 5 μL droplets. Baselines were assigned manually to minimise errors and measurements were taken at ambient temperatures across 10 different areas for each filter, with the reported contact angles being an average of these. Tilting angles were measured using 5 μL droplets and a digital protractor to obtain the angle at which droplets began to roll. Tilt angles were measured over water contact angle hysteresis, as the curvature of some of the samples prevented accurate measurement of static contact angles – due to obscuring of the wetting baselines. An Instron 68TM-10 (2 kN static load cell) with a 3-point flexure fixture was used to apply force to the samples (1 mm × 10 mm) to record the force required to fracture the samples. Abrasion durability was tested with sandpaper (grit 120) with a 100 g weight on top. Samples were moved 10 cm and then back, the cycle from back to forth was classed as one abrasion cycle (20 cm). Resistance to salinity was tested by preparing 3.5 wt/v% NaCl in DI water whilst pH durability was tested using solutions of KOH (pH12) or HCl (pH2) for 24 h. Samples were rinsed with DI water and dried at 80 °C with WCA measurements to follow.

The oil-water separation of the sintered silica was used to assess functionality. The separation mixture (100 mL), consisting of 1 : 1 water and chloroform was pipetted onto the top of the porous structure. Water was collected above the sample while the hydrophobic solvent passed through and collected in a beaker below. Chloroform volume was measured before and after separation to calculate the separation efficiency.

## 3. Results and discussion

The sintering method applied to the nanoparticles was aimed to offer a simple and relatively quick process to prepare porous structures by fusing particles together at temperatures below the melting point of the material. As the particles fuse, the overall grain size increases whilst the gap between particles shrinks producing small pores. Previously, borosilicate nanoparticles were used owing to their lower sintering temperature (∼900 °C) compared to silica nanoparticles (>1000 °C), however at these elevated temperatures silanol groups are removed (≡Si–OH + HO–Si≡ ⇔ ≡Si–O–Si≡ + H_2_O), leading to difficulty in functionalising the materials to induce high levels of hydrophobicity.^18^ LUDOX is an aqueous dispersion of colloidal silica, the received nanoparticles were manufactured using LUDOX-HS (40% wt SiO_2_) to achieve porous nanoparticles with a diameter ∼5 μm and a mean pore volume of 0.72 cm^2^ g^−1^.

**Fig. 2:**
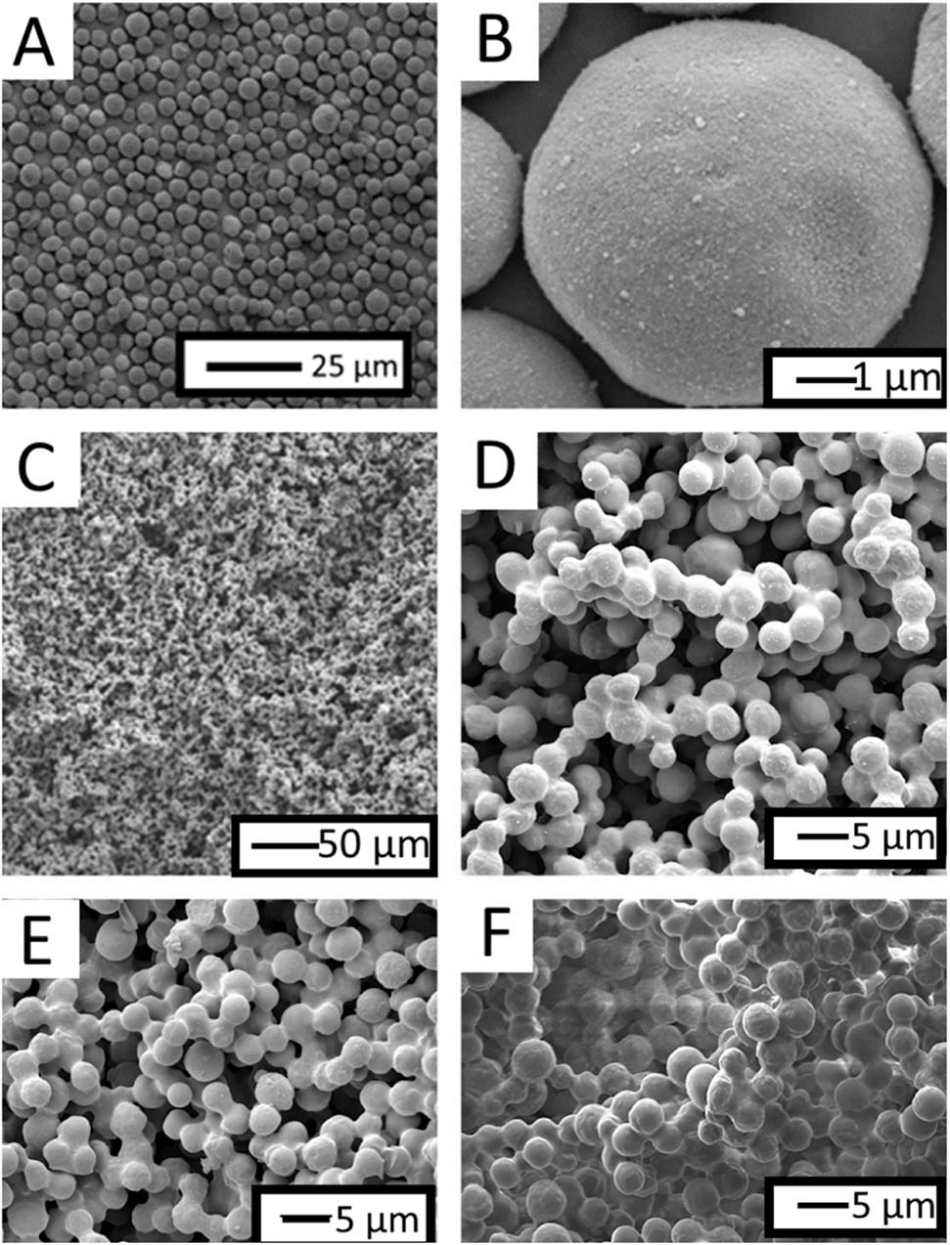
SEM micrographs of (A and B) as-received silica nanoparticles, and sintered particles at (C and D) 910 °C, (E) 945 °C, (F) 980 °C, highlighting the merging together of sintered particles to form larger agglomerates and less pores between particles.

Particles were sintered at a temperature of 910 °C or over and rehydoxylated in room temperature pH10 water before functionalisation using HMDS, as carried out using the same method attempted on the borosilicate samples. After hydrophobic modification, the produced samples showed superhydrophobic behaviour (Table 1). The surface morphology can be seen in Fig. 2C and D, where a rough microstructure of interconnected pores formed from the fusing of silica particles. As the sintering temperature was increased to 945 °C and 980 °C a thicker ‘neck’ region could be created (Fig. 2E and F) between particles and the influence this would additionally have on the WCA. At increased temperatures a larger number of particles formed through the merging together of multiple particles compared to a sintering temperature of 910 °C, leading to this increased neck radius (Fig. S1†) and shrinkage of interparticle distance (Fig. 2).

**Table 1:** Water contact and tilt angles at varying sintering temperatures

Sintering temperature (°C)	Water contact angle (°)	Tilt angle (°)
910	153	<5
945	153	<5
980	151	8

FTIR was used to assess if the functionalisation had been successful (Fig. S2†). Prior to sintering nanoparticles showed a broad peak ∼3400 cm^−1^ representative of –OH stretching. After sintering this peak remained, unlike in our previous work using milled borosilicate which showed the disappearance of the –OH peak (Fig. S3†) suggesting dehydration had occurred. FTIR analysis was undertaken after functionalisation with HMDS where the broad peak disappeared as the group was replaced with TMS. The wettability was then assessed with static water contact angle measurements. Despite these slight differences in morphologies, the WCAs remained consistent, with only samples prepared at 980 °C showing a tilt angle >5 °C.

**Fig. 3:**
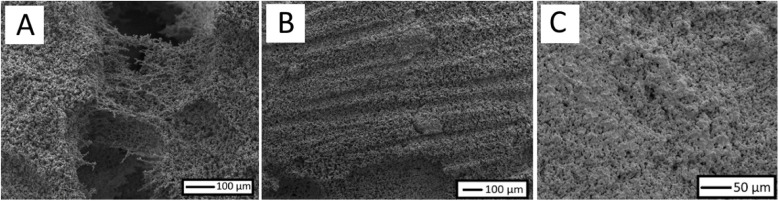
SEM micrographs of (A) regions of splitting between particles formed at sintering temperatures of 980 °C, (B) sintered samples (945 °C) after sandpaper abrasion, showing uneven removal of particles and (C) sintered samples (945 °C) after 24 h submersion in HCl (pH2).

The durability of the sample was tested for both mechanical (3-point bending) and chemical resistance. Each sintered sample (1 mm × 10 mm) was exerted to flexural stress until fracture, with the maximum stress being recorded at 0.28 MPa, 0.33 MPa and 0.27 MPa for sintering temperatures of 910 °C, 945 °C and 980 °C respectively. At 945 °C, the thicker regions between particles formed during the sintering process impart a greater strength onto the structure leading to an increased maximum stress compared to the thinner neck regions formed at 910 °C. Despite samples prepared at 945 °C having the largest neck regions and the greatest amount of particle merging, these samples showed the lowest maximum stress of the three sintering temperatures. Upon visualising the samples with SEM (prior to 3-point bending) it was noticed that relatively large regions of splitting had formed within the centre of the structures (Fig. 3A), these splits likely formed during the cooling of the structure and acted as points of weakness.

It is well known that the durability of superhydrophobic materials, as a result of the fragile micro/nanostructures, is one of their limiting features. In an aqueous environment with fluid flow, particles within the fluid will likely come into contact with the surface, possibly damaging the microstructure and changing wettability in the process. Sandpaper was used to abrade the surface to see the effect on the overall wettability, with the WCA angle being measured after each cycle. Though the sample remained superhydrophobic for the first few cycles (WCA > 150° up to cycle 3), the WCA eventually began to drop off, measuring in at 128° by cycle 10 (Fig. S4†). Additionally, the degree of variation in the measurements greatly increased with each additional cycle due to the uneven removal of the coating. SEM visualisation in Fig. 3B highlights this. Each scratch in the material reveals part of the unfunctionalized internal structure, as a result, even as roughness is maintained, hydrophilic surface chemistry is continually exposed which reduces the measured water contact angles.

To understand the durability against different chemical conditions, the resilience to salinity (3.5 wt/v%), acidity (pH2) and alkalinity (pH12) were tested by submerging samples in each solution for 24 hours. A salinity of 3.5 wt/v% was used to simulate the average salinity of the sea and after drying no noticeable difference was observed in the measured WCA. Unfortunately, for the alkaline conditions after drying samples became hydrophilic with WCAs being unable to be measured as the water passed through the surface porosity. The high pH likely led to the rehydroxylation of surface atoms, in a similar way that it was used to initially rehydroxylate particles before functionalisation. If these materials were to be used in a highly alkaline environment alternative techniques to induce hydrophobicity would need to be explored, such as coating with polymeric solutions. For the acidic test, the measured WCA showed a slight decrease down to 150 ± 6° and across the measurements, regions of superhydrophobicity and hydrophobicity (<150°) were observed. SEM images after submersion in acidic conditions (Fig. 3C) showed that regions had formed on the surface of multiple particles joining together altering the morphology of the surface. Though not large, these regions could explain the difference in wettability across the sample.

The functionality was finally assessed through oil/water separation tests. Water (hydrophilic), dyed blue with methylene blue, and hexane (hydrophobic), dyed with Nile red, were mixed together and slowly poured through the porous structure. Due to the superhydrophobic effect, the water was unable to pass through the porous structure, instead being collected above (horizontal position) or rolling off (tilted position) to be collected. The hydrophobic component could easily pass through the porous network. For small volumes, the oil would be completely absorbed and collected within the structure (Movie S1†), at larger volumes and as the structure became saturated, the oil would begin to pass through the pores to be collected below. The separation efficiency 
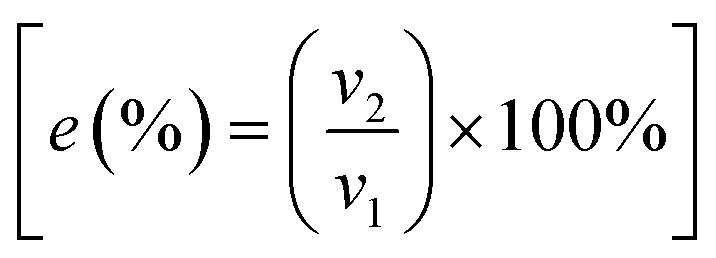
 (where *v*_1_ is the initial volume of hydrophobic solvent and *v*_2_ the collected volume) was calculated to be between 96–98% (Table S1†), (with the higher efficiency occurring for the lower sintering temperatures), which is as effective as our previous work on oil/water separation filters^19,20^ As shown in Movie S1,† the oil absorbs into the porous structure and as such is not all collected into the beaker to be measured, this will ultimately lead to a lower calculated separation efficiency. After oil/water separation the WCA's were measured again, and regions of decreased hydrophobicity were observed with an average WCA of 137 ± 12°. This results from the solvent trapped within the porosity, upon drying WCA's returned to >150° with no noticeable difference from samples before oil/water separation. Samples were additionally left in ambient conditions free of light for ∼3 months to assess the long term hydrophobicity, again no noticeable difference in WCA was measured.

## 4. Conclusions

Superhydrophobic materials were successfully prepared by sintering silica nanoparticles, achieving WCA's up to 153°. These silica particles offer the benefit of being made from commercially available nanoparticles and nanoparticle precursors. This is a proof-of-concept piece of work for future material fabrication where particle size can be altered to offer additional change to the porosity as required. The sintering technique allows for structures to be made in any shape depending on the desired use, for example, pipes could be created by inserting a mandrel within a pipe, pouring in powder, sintering and then removing the mandrel to achieve a hollow structure. Through changing the sintering temperature, pore size and structural strength can be altered, with increasing temperatures leading to larger neck regions, however, upon cooling of high temperature sintering, splits can form between particles leading to areas of weakness. The optimal sintering temperature within was 945 °C, demonstrating a maximal flexural tension of 0.33 MPa. Though the materials prove suitable for marine environments, not affected by a salinity of 3.5 wt/v%. There was a resilience against abrasion and only a partial resilience to acid/alkaline conditions, which requires improvement – possibly through alternative functionalisation routes. The prepared samples demonstrated the presence of plastrons when submerged, in addition to durability improvements future work can therefore focus on the implementation of pneumatic air control and monitoring of plastron lifetime.

## Author contributions

Conceptualization, ES and CRC; methodology, ES and CRC; validation, ES; formal analysis, ES; investigation, ES; resources, ES and CRC; data curation, ES and CRC; writing – original draft preparation, review and editing ES and CRC; supervision, CRC; project administration, CRC; funding acquisition, CRC.

## Conflicts of interest

The authors declare no conflict of interest.

## Supplementary Material

RA-014-D4RA02534B-s001

RA-014-D4RA02534B-s002

RA-014-D4RA02534B-s003

## References

[cit1] Xue Y., Lv P., Lin H., Duan H. (2016). Appl. Mech. Rev..

[cit2] Mehanna Y. A., Sadler E., Upton R. L., Kempchinsky A. G., Lu Y., Crick C. R. (2021). Chem. Soc. Rev..

[cit3] Duan H. (2017). Procedia Appl. Mech. Rev..

[cit4] Lee C., Choi C. H., Kim C. J. (2016). Exp. Fluids.

[cit5] Liravi M., Pakzad H., Moosavi A., Nouri-Borujerdi A. (2020). Prog. Org. Coat..

[cit6] wei Wang X., ye Fan Z., qi Tang Z., Jiang N. (2021). J. Hydrodyn..

[cit7] Gose J. W., Golovin K., Boban M., Mabry J. M., Tuteja A., Perlin M., Ceccio S. L. (2018). J. Fluid Mech..

[cit8] Ling H., Srinivasan S., Golovin K., McKinley G. H., Tuteja A., Katz J. (2016). J. Fluid Mech..

[cit9] Park H., Sun G., Kim C. J. (2014). J. Fluid Mech..

[cit10] Aljallis E., Sarshar M. A., Datla R., Sikka V., Jones A., Choi C. H. (2013). Phys. Fluids.

[cit11] Kavalenka M. N., Vüllers F., Lischker S., Zeiger C., Hopf A., Röhrig M., Rapp B. E., Worgull M., Hölscher H. (2015). ACS Appl. Mater. Interfaces.

[cit12] Panter J. R., Kusumaatmaja H. (2017). J. Phys.: Condens. Matter.

[cit13] Vüllers F., Germain Y., Petit L. M., Hölscher H., Kavalenka M. N. (2018). Adv. Mater. Interfaces.

[cit14] Han X., Liu J., Wang M., Upmanyu M., Wang H. (2022). ACS Appl. Mater. Interfaces.

[cit15] Choi W., Kang M., Park J. Y., Jeong H. E., Lee S. J. (2021). Phys. Fluids.

[cit16] Kirkland J. J. and Köhler J., Porous Silica Microspheres Having Silanol-Enriched and Silanized Surfaces, 1989

[cit17] Kern W., Puotinend D. (1970). RCA Rev..

[cit18] Wan Q., Ramsey C., Baran G. (2010). J. Therm. Anal. Calorim..

[cit19] Mehanna Y. A., Crick C. R. (2020). Materials.

[cit20] Sadler E., Crick C. R. (2021). Sustainable Mater. Technol..

