# Supplementary material for: The fabrication of robust and highly efficient oil–water separation filters via the high temperature sintering of silica micropower
Source: RSC Adv. 2024 Apr 25;14(19):13489–93. doi: 10.1039/d4ra02534b (PMC11043794; doi:10.1039/d4ra02534b)
Supplement: RA-014-D4RA02534B-s003 [file RA-014-D4RA02534B-s003.pdf]

Electronic Supplementary Material (ESI) for RSC Advances.
This journal is © The Royal Society of Chemistry 2024

The Fabrication of Robust and Highly Efficient Oil-water Separation Filters *via* the High Temperature Sintering of Silica Micropower

Electronic supplementary material (ESI†)

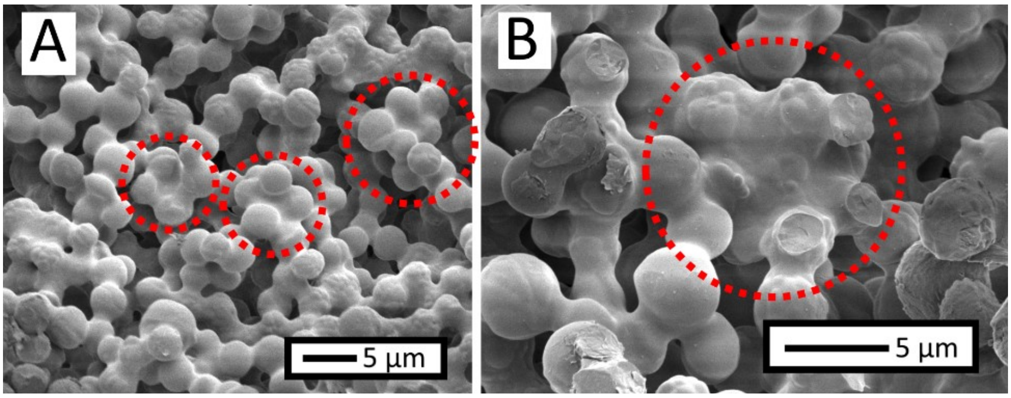

Figure S1. SEM micrographs of a surface showing the merging together of multiple particles at sintering temperatures of A) 945 ºC, and B) 980 ºC.

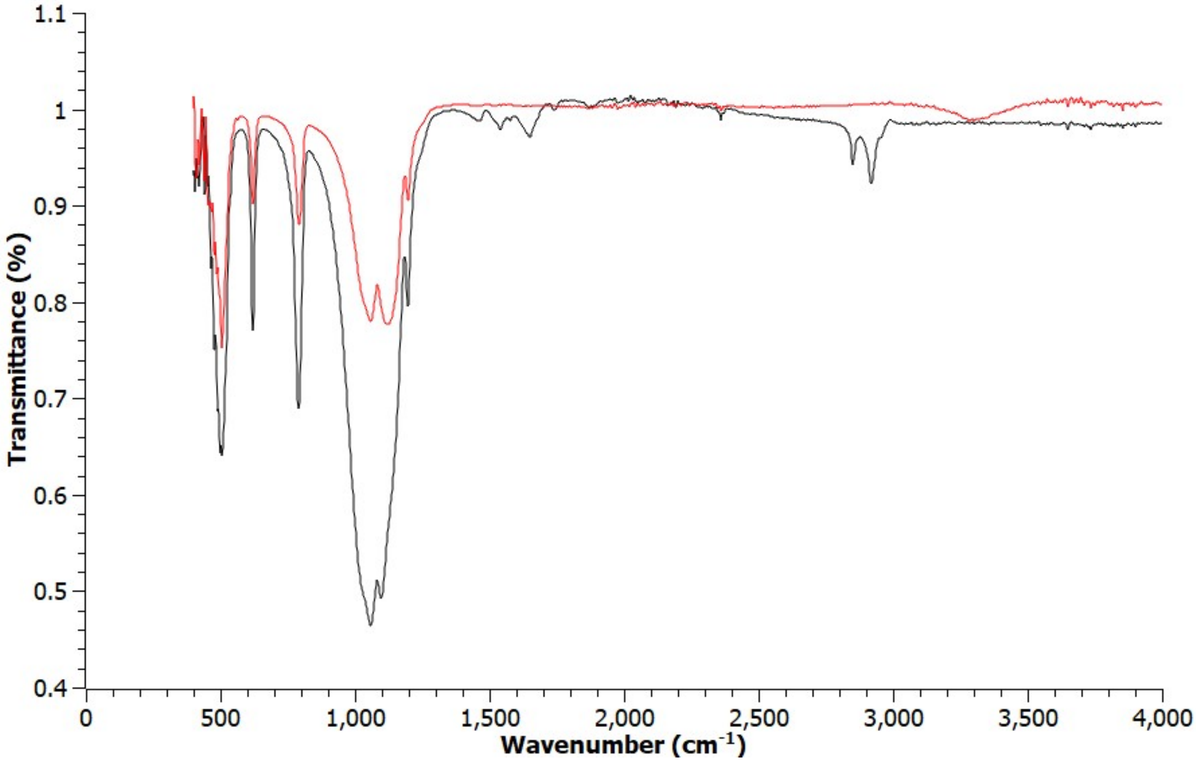

Figure S2 FTIR spectra of sintered silica nanoparticles before (red) and after (black) HMDS functionalisation.

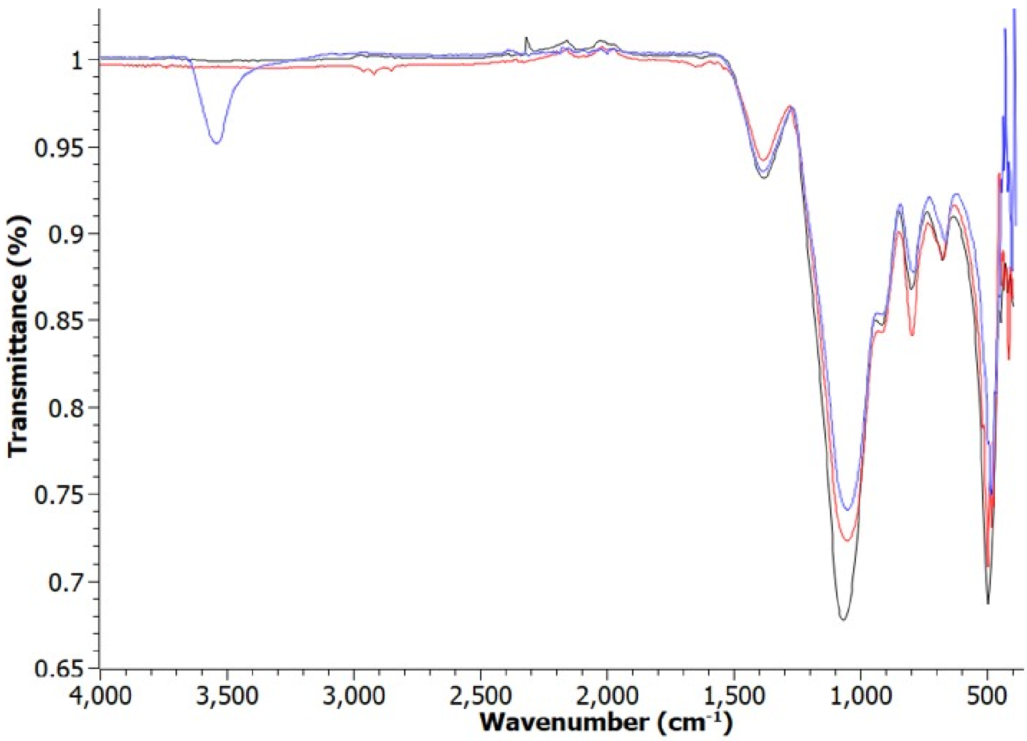

Figure S3 FTIR spectra of blue) as received borosilicate, black) Sintered borosilicate and red) after attempted functionalisation with HMDS. Notably, after sintering the -OH peak (~3500 cm^{-1}) disappears suggesting that dehydration occurred at the high sintering temperatures. Small peaks at ~2940 cm^{-1} are indicative of CH_3 stretches on the HMDS functionalised sample.

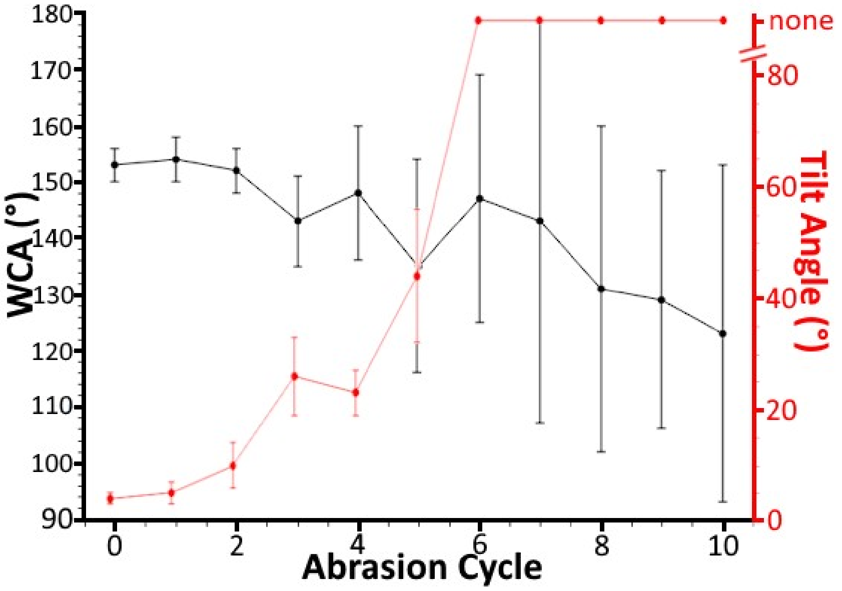

Figure S4 Average WCA and Tilt angles after sandpaper abrasions.

Sintering Temperature (°C)	Collected Volume (mL)	Separation Efficiency (%)
910	49.0	98.0
945	48.2	96.4
980	47.8	95.6

Table S1 Oil/water Separation data for the separation of 1:1 mixture consisting of water (50 mL) and chloroform (50 mL). Separation efficiency is calculated following [$e(\%) = \left(\frac{v_2}{v_1}\right) x 100\%$] where v_1 is the initial volume of hydrophobic solvent and v_2 the collected volume.
